# Supplementary material for: The interactome of intact mitochondria by cross-linking mass spectrometry provides evidence for coexisting respiratory supercomplexes
Source: Mol Cell Proteomics. 2017 Dec 8;17(2):216–32. doi: 10.1074/mcp.RA117.000470 (PMC5795388; doi:10.1074/mcp.RA117.000470)
Supplement: Supplemental Data [file supp_RA117.000470_133922_0_supp_23978_fzffwf.zip › spectra_annotation/mito_spectra_annotation/21-1-3-1-4-1.pdf]

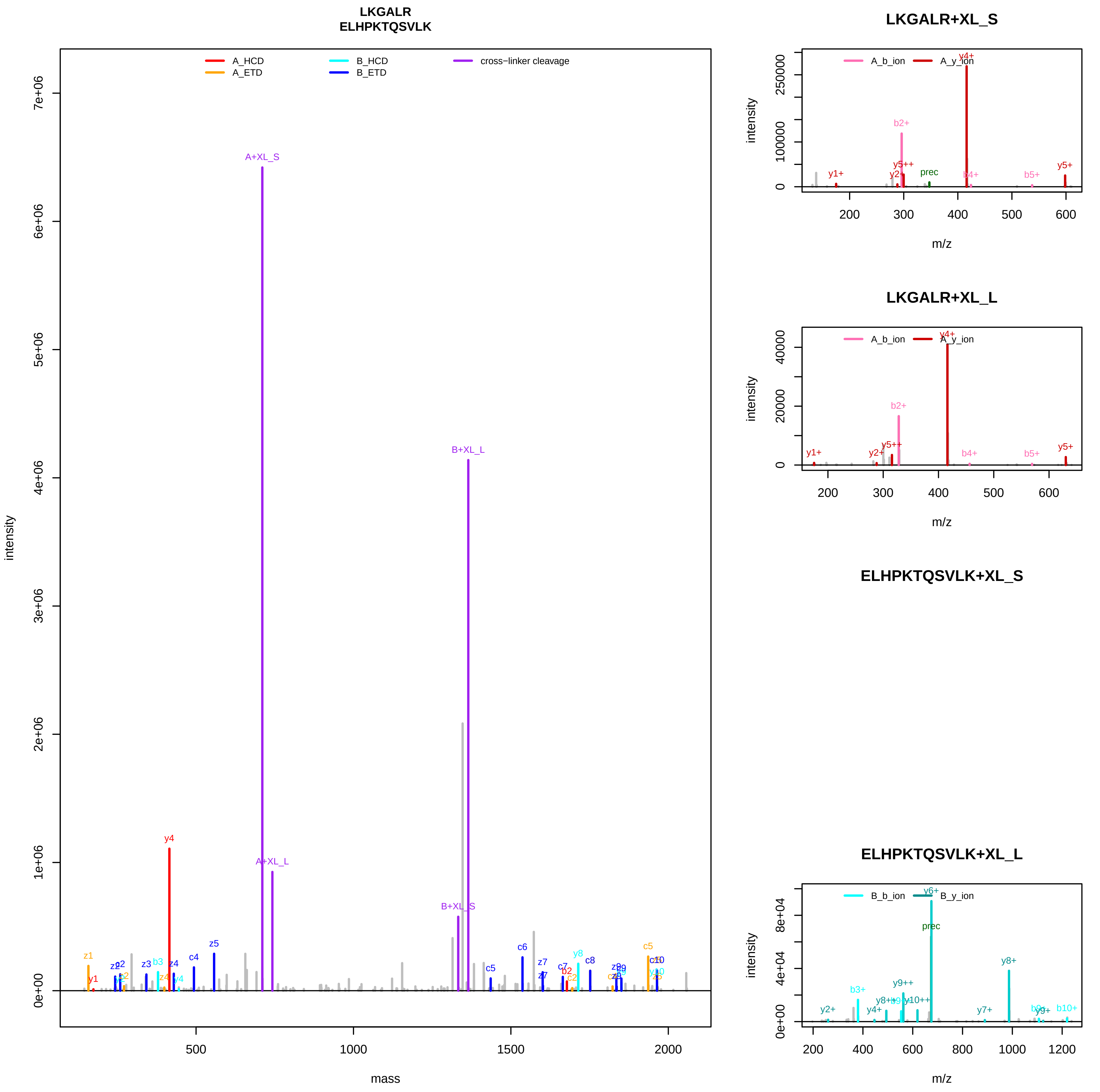

LKGALR
ELHPKTQSVLK
A_HCD
A_ETD
B_HCD
B_ETD
cross−linker cleavage
A+XL_S
B+XL_L
A+XL_L
B+XL_S
y4
z1
y1
z2
c2
z3
b3
z4
c4
z5
c5
c6
z7
c7
b2
y8
c8
c10
intensity
mass
0e+00
1e+06
2e+06
3e+06
4e+06
5e+06
6e+06
7e+06
500
1000
1500
2000
LKGALR+XL_S
A_b_ion
A_y_ion
y4+
b2+
y1+
y2+
y5++
prec
b4+
b5+
y5+
LKGALR+XL_L
ELHPKTQSVLK+XL_S
ELHPKTQSVLK+XL_L
B_b_ion
B_y_ion
y6+
prec
y8+
b3+
y9++
y8++
y10++
y2+
y4+
y7+
y9+
b10+
m/z
